# *In situ* Measurements of Phytoplankton Fluorescence Using Low Cost Electronics

**DOI:** 10.3390/s130607872

**Published:** 2013-06-19

**Authors:** Thomas Leeuw, Emmanuel S. Boss, Dana L. Wright

**Affiliations:** School of Marine Sciences, University of Maine, Orono, ME 04469, USA; E-Mails: emmanuel.boss@maine.edu (E.S.B.); dlwright2@alaska.edu (D.L.W.)

**Keywords:** fluorometer, fluorescence, phytoplankton, chlorophyll, Arduino, inexpensive, sensors, oceanography, technology, education

## Abstract

Chlorophyll *a* fluorometry has long been used as a method to study phytoplankton in the ocean. *In situ* fluorometry is used frequently in oceanography to provide depth-resolved estimates of phytoplankton biomass. However, the high price of commercially manufactured *in situ* fluorometers has made them unavailable to some individuals and institutions. Presented here is an investigation into building an *in situ* fluorometer using low cost electronics. The goal was to construct an easily reproducible *in situ* fluorometer from simple and widely available electronic components. The simplicity and modest cost of the sensor makes it valuable to students and professionals alike. Open source sharing of architecture and software will allow students to reconstruct and customize the sensor on a small budget. Research applications that require numerous *in situ* fluorometers or expendable fluorometers can also benefit from this study. The sensor costs US$150.00 and can be constructed with little to no previous experience. The sensor uses a blue LED to excite chlorophyll *a* and measures fluorescence using a silicon photodiode. The sensor is controlled by an Arduino microcontroller that also serves as a data logger.

## 1. Introduction

In oceanography, monitoring the distribution of phytoplankton (microscopic algae) in the water column is vital to understanding many large-scale physical and biological processes. Measurement of phytoplankton abundance in seawater is relatively simple due to the unique fluorescent properties of chlorophyll *a*, which is contained inside phytoplankton cells. The combination of chlorophyll *a* and other cellular components inside phytoplankton cells have a maximum absorption near 440 nm and maximum fluorescence at 685 nm [1]. The unique wavelength of fluorescence (685 nm) and spectral distance between excitation and emission wavelengths has made fluorescence a reliable proxy for phytoplankton biomass for over 50 years [2].

Historically, fluorescence was primarily measured *in vitro*. In the past few decades, advances in technology have allowed for high resolution *in situ* monitoring of phytoplankton biomass. Today, fluorescence of chlorophyll *a* can even be measured from aircraft or satellites [3,4]. However, aerial methods can only measure chlorophyll *a* at the ocean surface during daylight hours. High resolution measurements of chlorophyll *a* concentration at depth require the use of submersible *in situ* fluorometers. This makes *in situ* fluorometers an essential tool for 21st century oceanography.

The retail price of commercial *in situ* fluorometers (>US$3,000) has limited their availability to both students and professionals. This is especially true if the desired spatial resolution requires multiple fluorometers. However, recent technological advances have reduced the size and price of many electronic components used to build optical sensors [5,6].

This study demonstrates the use of low cost electronics to measure *in situ* phytoplankton fluorescence. The goal was to construct a cost effective *in situ* fluorometer that could be easily reproduced by individuals with no prior engineering background. Ideally, this design would allow anyone with US$150.00 to collect accurate measurements of *in situ* phytoplankton fluorescence. Similar low cost sensors have been constructed in the past. For example, Schofield [7] constructed a fluorometer using low cost components to measure DNA marked with fluorescent tags. Low cost optical sensors have also been used in numerous biomedical applications [8]. However, these sensors were designed primarily for lab use. This study attempts to bring low cost instrumentation out of the lab and into the field.

## 2. Methods

**Figure 1.**
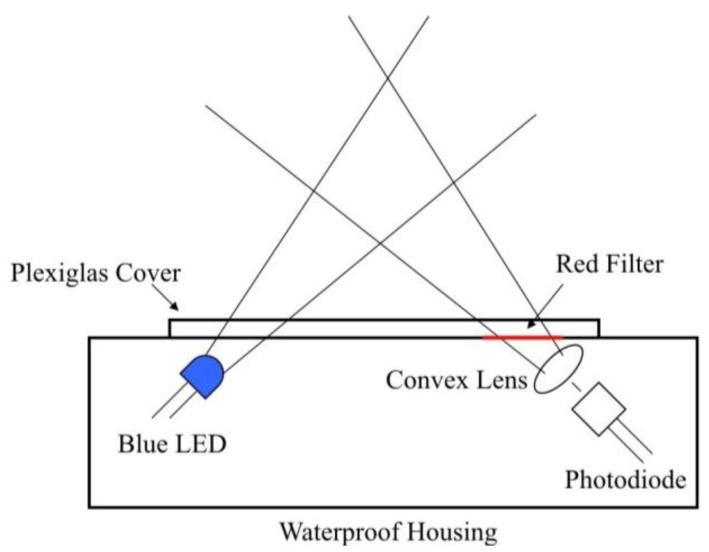
Sensor components and geometry. The detector is placed at 90° to the light source to minimize the amount of excitation light scattered toward the detector.

Like many commercially manufactured *in situ* fluorometers, the sensor uses a flat face design. Two holes were drilled through the flat side of a waterproof case at a 45° angle (Figure 1). These two holes provided a window for light to pass through the opaque housing. The holes were covered with a square of ¼ inch (0.635 cm) Plexiglas^®^ to keep the housing watertight while still providing an optically clear window. The Plexiglas^®^ was adhered to the housing using IPS Weld-On 3 (Compton, CA, USA). The light source and detector were mounted just behind the two 45° angle holes such that the angle between the cone of illumination and cone of detection was on average 90°. This helped to reduce the amount of excitation light scattered toward the detector. The flat face design used here is ideal because it requires minimal alterations to prefabricated waterproof cases.

**Figure 2.**
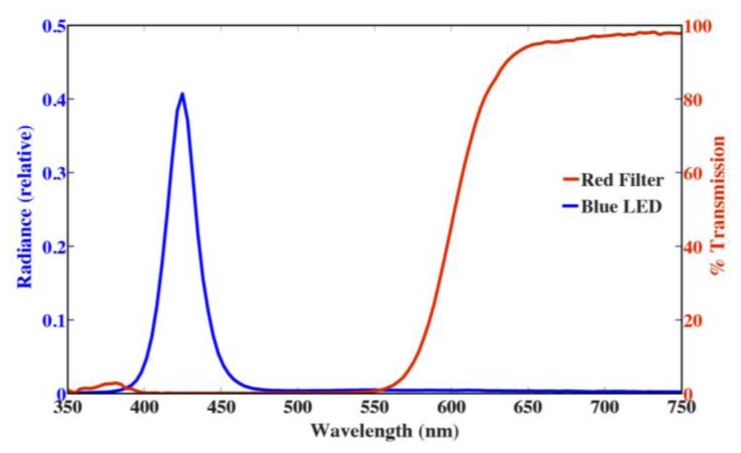
Emission spectrum from LED420L and percent transmission of Roscolux filter #19.

A blue LED with a ball lens was used as the excitation light source. The beam angle of the LED was 18°. This is wider then necessary, however it allowed for easier alignment of the light source and detector. The LED emits a narrow band of light centered at 425 nm (Figure 2). The LED was wired in parallel with two 10 Ω resistors. This provided a current of 85 mA when supplied with 5 V. The LED was powered by one of the microcontroller digital pins (described below), which allowed it to be turned on and off in the microcontroller script. Both the maximum wavelength of emission and the output of the LED over time were characterized using a Satlantic HyperOCR radiometer (Halifax, NS, Canada).

The detector consisted of a red filter, convex lens, photodiode, and amplification circuit. The light detector itself was a silicon FDS 100 photodiode. The FDS 100 is sensitive to all visible wavelengths of light. Therefore, a Roscolux #19 filter (Rosco, Stamford, CT, USA) was placed over the photodiode to shield it from the blue excitation light (Figure 2). The lens helped to collect fluoresced light and focus it on the small face of the photodiode.

**Figure 3.**
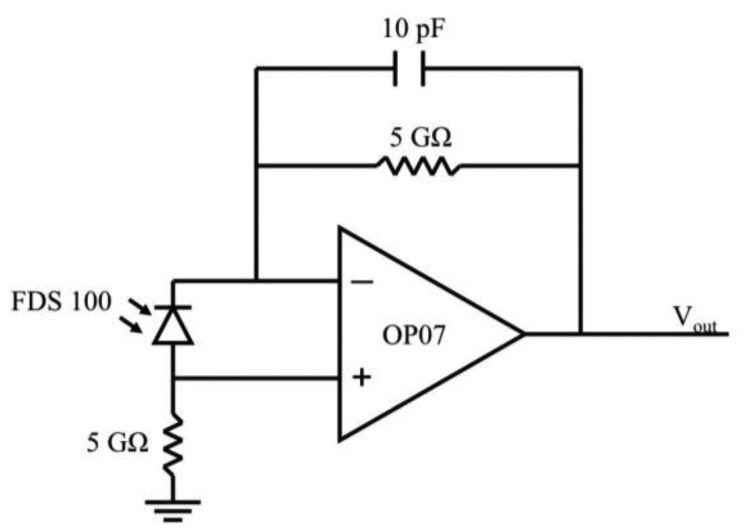
Transimpedance amplifier using OP07 and 5 GΩ resistors.

The current produced by the photodiode was amplified using a transimpedance amplifier. The gain of this amplifier is equal to the sum of the two resistors used in the circuit (Figure 3). The resistance values can be adjusted if the specific application requires a detector that has greater or lesser sensitivity to light. The transimpedance circuit not only amplifies, but also converts the current produced by the photodiode to a voltage that can be read at V_out_ (Figure 3). The transimpedance circuit requires very few components and can be construed with minimal soldering.

The sensor was controlled using an Arduino Duemilanove (ATmega328) microcontroller (Smart Projects, Strambino, Italy). Two 9 V batteries connected in parallel were used to power the Arduino, which in turn provided power to the LED and amplification circuit. The Arduino was also used to set the sampling interval of the sensor (time between measurements) by turning the LED and amplification circuit on and off (see Supplementary Material for the Arduino code).

An SD card attachment for the Arduino was used to log data collected by the sensor. To record the output from the detector, one of the Arduino analog pins was connected to the output of the amplifier (V_out_ in Figure 3). The Arduino analog pins are capable of measuring voltages between 0 and 5 V, with a 10-bit resolution. The Arduino analog to digital converter measures voltage in counts ranging from 0 to 1,023 (∼0.005 V/count). Voltage, measured in counts, was stored on the SD card along with the date and time of each measurement.

The completed sensor was calibrated using both extracted chlorophyll *a* from spinach leaves and with live phytoplankton cells. The sensor was cross calibrated with a WetLabs WETStar chlorophyll *a* fluorometer (Philomath, OR, USA). Calibration with extracted chlorophyll *a* was conducted in 25 °C deionized water. Calibration with live cells was conducted in 17 °C filtered seawater using the phytoplankton *Thalassiosira weissflogii*. Aliquots from a stock mixture of extracted chlorophyll *a* or *T*. *weissflogii* were added to approximately 9 L of water. Both the constructed fluorometer and a WETStar fluorometer were immersed in the mixture. Type one linear regression between the output of the constructed fluorometer and the WETStar fluorometer yielded the relationship between counts and chlorophyll *a* concentration.

The sensor was also tested to determine its response to water turbidity. A mixture of 0.8 g of clay and 1 L of deionized water were mixed thoroughly. Aliquots from the 1 L mixture were sequentially added to 9 L of deionized water to create clay concentrations from 10 to 80 g·m^−3^. The sensor was immersed in each dilution and allowed to collect 10 readings.

Two *in situ* overnight deployments of the sensor in the Damariscotta River Estuary were conducted at the Darling Marine Center (DMC), located in Walpole, ME, USA. The first deployment was conducted on 3 April 2012. The sensor was suspended off the floating dock at the DMC at a depth of two meters. The sensor collected 10 measurements every 10 minutes until it was recovered the following morning. The second deployment was conducted on 1 August 2012. The sensor was deployed in conjunction with a Seabird CTD off the floating dock at the DMC. The CTD was outfitted with the same WETStar fluorometer used to cross-calibrate the sensor. The CTD and constructed fluorometer were recovered the morning of 2 August 2012. During the deployment water samples were collected next to the CTD using a niskin bottle. The water samples were used to measure extracted chlorophyll *a* according to the JGOFS protocol [9].

## 3. Results and Discussion

### 3.1. Components and Price

The total price of the sensor was US$143.04, not including shipping and handling costs (Table 1). The most expensive component of the sensor was the blue LED light source. Different waterproof containers could also be purchased, depending on size and depth requirements. The container used to house the sensor in this example was larger then necessary. The housing was sufficient for shallow (<5 m) deployments for multiple days at a time. A more robust housing will likely be required for deeper and/or longer deployments.

### 3.2. Laboratory Test

**Figure 4.**
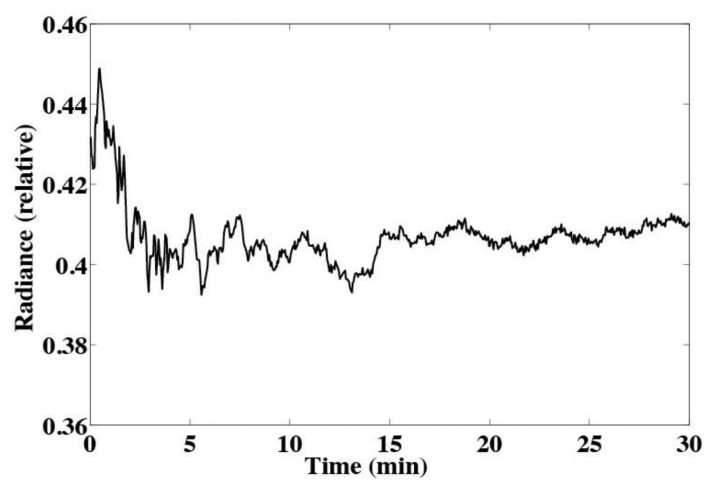
Radiance at 425 nm of blue LED over time. The LED should be allowed to run for at least 1.5 minutes to allow the output to stabilize.

When power is first applied to a light source, the output can be unstable. Numerous optical instruments cannot be used before warming up the light source. The output of the LED used in this design appears slightly unstable when power is first applied (Figure 4). Therefore, the LED was allowed to run for 1.5 minutes before each measurement. This allowed the output of the LED to stabilize before each measurement was made. The amount of time the LED is allowed to warm up is a trade off between light source stability and battery life. If measurements must be made less then 1.5 minutes apart, the LED should be allowed to run continuously.

Using two 9 V batteries the sensor was able to log data for approximately 24 hours (with a sampling interval of 10 minutes and allowing the LED to warm up for 1.5 minutes before each sample). When deploying the sensor for an extended period of time, it is imperative that it is powered by an appropriately sized battery pack. If the voltage supplied to the Arduino drops below 6 V, data on the SD card can be lost or become corrupted.

**Figure 5.**
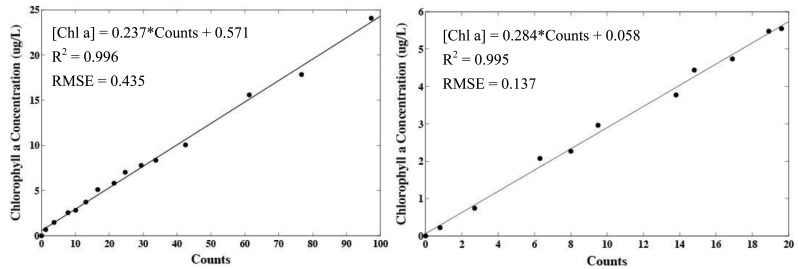
Cross calibration using extracted chlorophyll *a* from spinach leaves (**Left**) and live *Thalassiosira weissflogii* cells (**Right**). Regression equation, coefficient of determination, and root mean squared error (RMSE) are included in each plot.

Cross calibration with a WetLabs WETStar fluorometer showed the linear dependence of counts on chlorophyll *a* concentration. The type one least-squares linear regressions for both calibrations have an R^2^ > 0.99 and p < 0.001 showing a significant positive correlation with chlorophyll *a* concentration (Figure 5). The mean absolute error for the extracted and *in vivo* calibrations was 0.34 and 0.13 μg·L^−1^, respectively (assuming the WETStar flurometer was well calibrated). The absolute error generally increased with increasing chlorophyll *a* concentration. For larger chlorophyll *a* concentrations (>2 μg·L^−1^) the relative error was on average ±4 % of the signal.

The slope of the regression and mean error for the extracted chlorophyll *a* calibration is larger than for the *in vivo* calibration. This is likely a result of the shifted excitation and emission peaks when chlorophyll *a* is dissolved in an organic solvent [10]. The WETStar fluorometer excitation light (∼460 nm) is also not optimized to excite extracted chlorophyll *a*. However, this regression analysis only demonstrates the linear response of the sensor and is not intended as an absolute calibration. The fluorescence yield of phytoplankton is highly influenced by the species composition, photoadaptive state, and physiological state [11]. Thus, it is typical to collect water samples near the area being monitored for measurement of extracted chlorophyll *a*. The extracted chlorophyll *a* measurements are then used to calibrate the sensor under the current environmental conditions (field calibration). However, if no field calibration is conducted, the signal is still useful as a measure of variability in chlorophyll *a* concentration.

**Figure 6.**
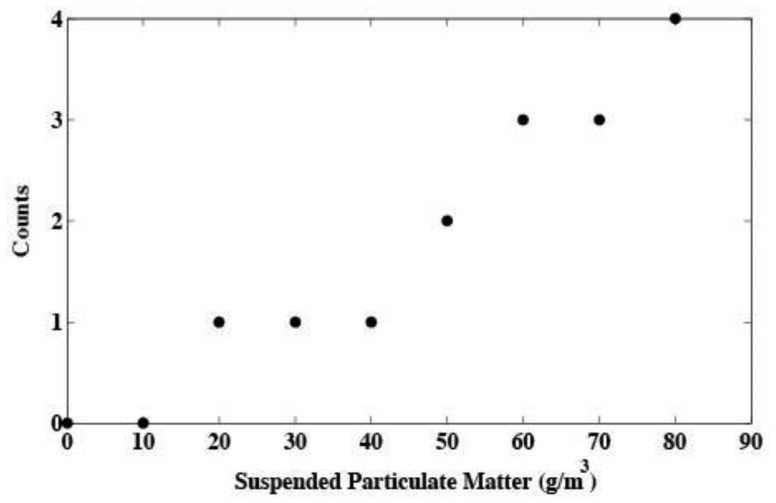
Sensor response to water turbidity. The figure shows counts returned by the sensor when placed in a mixture of deionized water and varying amounts of bentonite clay.

Highly turbid water can introduce error into fluorescence measurements by scattering the excitation light toward the detector. To study the magnitude of this effect, the output from the sensor was monitored while immersed in varying concentrations of suspended particulate matter. Turbidity was found to introduce a slight error of up to four counts in extremely turbid water (Figure 6). However, the amount of suspended material in many environments rarely exceeds 50 g·m^−3^ [12,13]. Concentrations below 50 g ·m^−3^ introduce an error on the order of one count as result of turbidity. Using the calibration in Figure 5 on *T*. *weissflogii*, one count corresponds to approximately 0.28 μg·L^−1^ of chlorophyll *a*.

### 3.3. Field Deployments

**Figure 7.**
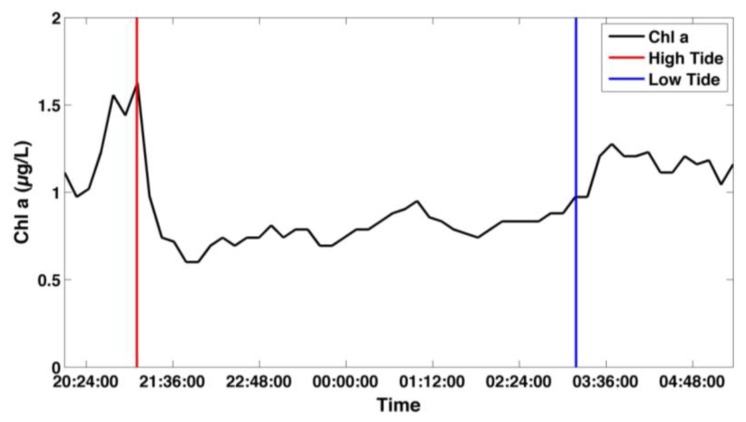
Time series of chlorophyll *a* concentration measured in the Damariscotta River Estuary during first deployment (04/03/12–04/04/12). Vertical lines show time of high and low tide.

During the first deployment, the sensor successfully logged over 500 chlorophyll *a* readings. The 10 measurements taken every 10 minutes were median binned. The laboratory calibration using *T. weissflogii* was applied to the sensor output since no extracted chlorophyll *a* measurements were taken. Tidal fluctuations in chlorophyll *a* can be seen in the chlorophyll *a* time series (Figure 7). These fluctuations are typical of Damariscotta River Estuary [14], however, there was no validation of the data collected during the first deployment.

**Figure 8.**
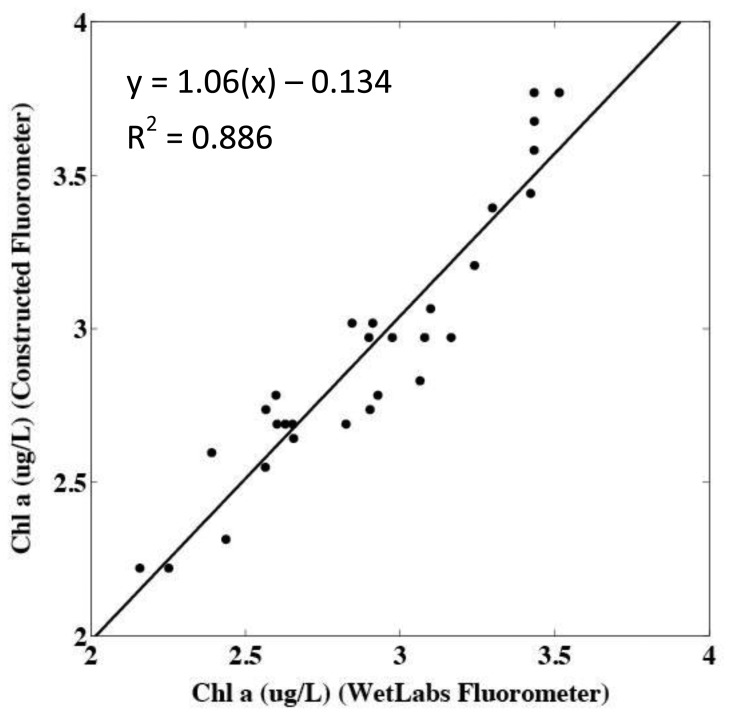
Comparison of chlorophyll *a* concentration measured by WetLabs WETStar and the constructed fluorometer in The Damariscotta River Estuary (08/01/12–08/02/12). The outputs from both sensors were normalized to extracted chlorophyll *a* measurements taken from water collected during the deployment.

The second deployment was in conjunction with a CTD and the WETStar fluorometer used in the initial calibration of the sensor. The extracted chlorophyll *a* measurements taken during the deployment were used to calibrate both sensors to the current environmental conditions. During the first half of the deployment, output from the constructed sensor appeared noisy and did not correlate well with the WETStar fluorometer. This may have been due to interference between the constructed sensor and the CTD pump which were located close together. During the second half of the deployment the sampling cycle of the CTD and the constructed fluorometer became slightly offset. Data collected by the sensor during the second half the deployment were not noisy and correlated well with the WETStar fluorometer (Figure 8).

### 3.4. Improvements

There are numerous improvements that could make the sensor more versatile and more accurate. The primary limitation of the sensor is sensitivity to ambient light. The detector can easily become saturated by ambient light, thus ambient light rejection will be required for daytime operation near the water surface. This can be done using two methods. The first is to modulate the light source and use a high frequency filter as part of the detection circuit. Ambient light tends to change slowly, making the signal from ambient light low frequency (with the exception of focusing and defocusing of capillary waves). Modulation of the light source will cause light from florescence to reach the detector in high frequency pulses. A high frequency filter will allow the high frequency signal through while blocking low frequency signals. Modulation of the light source can be accomplished using the Arduino microcontroller. The Arduino has built in digital pins for pulse width modulation (PWM). These can be used to drive an LED at high frequencies. Digital pins can also be switched from 0 to 5 V at any given frequency. This allows for customization of both the frequency and duty cycle of the LED. This method of ambient light rejection is used in many optical communication devices. However, this will significantly increase the complexity of the sensor.

A simpler solution is to convert the sensor to a flow through system, where the measurement is made inside a tube that excludes ambient light. The conversion of the sensor to a flow through system is relatively simple. A piece of opaque rigid tubing could be placed over the face of the sensor or an acrylic tube can be passed through the center of housing. This is the recommended configuration for daylight operation, and would add only a few dollars to the total price of the sensor.

If placed in close proximity to a pump or other source of electromagnetic field, the sensor may need to be shielded to prevent noise. The sensor can be shielded from electromagnetic fields by placing the sensor inside a faraday cage. Lining the housing with tin foil and connecting the foil to the ground pin on the Arduino would provide adequate shielding.

Studying the effects of temperature on the sensor will also help to improve performance. Temperature does affect semiconductors, such as LEDs and photodiodes, however the effect is predictable [15]. For short-term deployments, a field calibration of the sensor should account for temperature effects. For longer deployments, or vertical profiles spanning large temperature ranges, temperature may introduce a significant error. If this occurs, the output of the LED can be characterized over a wide range of temperatures. This information can be used to apply temperature corrections to the fluorescence signal. To measure the temperature inside the sensor housing, a thermistor (temperature sensitive resistor) could be placed inside the housing. The Arduino has numerous analog pins that could be used to read voltages from the thermistor. Voltages corresponding to temperature could be logged next to fluorescence data on the SD card. The addition of a thermistor and the required resistor would add less then US$1.00 to the price of the sensor.

## 4. Conclusions

During both laboratory calibrations and field tests, the constructed sensor demonstrated excellent linearity in response to chlorophyll *a* fluorescence. The error in the sensor measurement, in comparison to a commercial instrument, was approximately ±4 % of the signal (with a minimum error of 0.3 μg·L^−1^ for small values chlorophyll *a*). The range of chlorophyll *a* concentrations in the North Atlantic alone spans three orders of magnitude from 0.029 to 32.6 μg·L^−1^ [16]. In coastal areas chlorophyll *a* concentration can reach 90 μg·L^−1^ [14]. Thus an error of ±4 % in fluorescence relative to a commercial instrument is sufficiently accurate for many oceanographic and water quality monitoring applications. The resolution of the sensor is also well above the resolution needed for detecting phytoplankton blooms. Thus, the sensor could be used to detect and monitor the development of phytoplankton blooms on large spatial scales. Similarly it could be deployed in an array to aid in the detection of harmful algal blooms.

**Figure 9.**
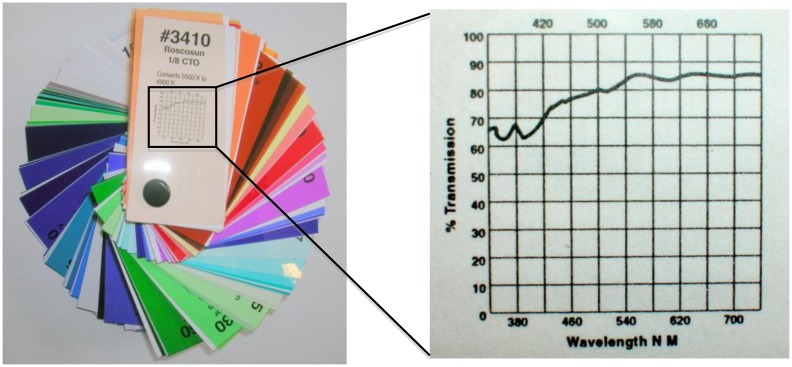
Roscolux filter booklet from Edmund Optics (included in the total price of the fluorometer). A booklet contains over 200 filters, each with a graph showing percent transmission. These can be used over the light source or detector to measure fluorescence from numerous other substances in addition to chlorophyll *a*.

There are numerous other molecules or compounds that are measured using fluorescence besides chlorophyll *a*. In oceanography fluorescence is also used to measure chromophoric dissolved organic material (CDOM) and other phytoplankton pigments such as phycoerythrin and phycocyanin. In addition to oceanography, fluorescence has application in microbiology, botany, chemistry, mineralogy, and geology [17,18]. Fluorescence dyes are also commonly used to measure flow rates of river and dispersion of waterborne toxins [19,20]. Interchangeable filters and light sources would allow the sensor to measure fluorescence from a number of materials (Figure 9). The resistor values can also be adjusted to change the sensitivity of the amplification circuit.

This sensor can also be used as an educational tool. A project where students build the sensor using the above components would provide students with an understanding of how fluorometers and related optical sensors work. It will also give students basic experience in engineering and programming. To further reduce the cost for educators, it can be built as a laboratory instrument. As a laboratory instrument there is no need for the waterproof housing, SD card, or data logger (the lens may also not be required).

Overall, this study has shown the potential for constructing low cost sensors for environmental applications. A fluorometer provides a good example because it is a moderately complex sensor. Other sensors used in oceanography can be construed using even fewer components. For example, sensors to measure temperature, optical scattering, photosynthetically active radiation (PAR), depth, and/or light attenuation could be constructed using even simpler techniques. Therefore, one could construct a whole array of sensors at little cost.

## Supplementary Material



## Figures and Tables

**Table 1.** List of prices (in US dollars) and sources for all components in the sensor.

**Component**	**Source**	**Price**
Blue LED (LED420L)	ThorLabs	$28.49
Waterproof Box (Drybox 2500)	Otterbox	$20.49
Arduino Duemilanove	Amazon	$20.00
1/2″Convex Lens (f = 15 mm)	ThorLabs	$19.70
Photodiode (FDS100)	ThorLabs	$13.10
(2) 5 GΩ Resistor	Digi-Key	$10.20
Roscolux Filter Booklet	Edmund Optics	$9.70
SD Card Shield (Data Logger)	imall.iteadstudio.com	$9.50
2 GB SD Card	Amazon	$5.29
Operational Amplifier (OP07)	Digi-Key	$4.04
Printed Circuit Board	RadioShack	$2.00
(2) 10 Ω Resistor	Digi-Key	$0.24
10 pF Capacitor	Digi-Key	$0.29

**Total**		**$143.04**
