# Supplementary material for: In situ Measurements of Phytoplankton Fluorescence Using Low Cost Electronics
Source: Sensors (Basel). 2013 Jun 19;13(6):7872–83. doi: 10.3390/s130607872 (PMC3715229; doi:10.3390/s130607872)
Supplement: Supplementary file 1 [file sensors-13-07872-s001.pdf]

OPEN ACCESS
sensors
ISSN 1424-8220
www.mdpi.com/journal/sensors

Supplementary Information

In situ Measurements of Phytoplankton Fluorescence Using Low Cost Electronics. *Sensors* 2013, *13*, 7882-7883

Thomas Leeuw *, Emmanuel S. Boss and Dana L. Wright

School of Marine Sciences, University of Maine, Orono, ME 04469, USA;
E-Mails: emmanuel.boss@maine.edu (E.S.B.); dlwright2@alaska.edu (D.L.W.)

* Author to whom correspondence should be addressed; E-Mail: thomas.leeuw@umit.maine.edu;
Tel.: +1-207-610-3732.

Arduino Code for Low Cost Fluorometer

The Arduino is programmed using a language similar to C/C++. The script used to run the sensor requires two additional libraries (SD and Time) that are available online. Prior to running the sensor, a text file must be saved to the SD card. The name of the text file must correspond to the one found in the Arduino script. In this example the file is named 'data.txt.' The script used for the sensor is provided below (note the LED is connected to digital pin 4 and the output from the amplifier is read on analog pin 0):

```
#include "SD.h"
#include "Time.h"
int read1;
int read2;
int read3;
int read4;
int read5;
int val;
File myFile;
void setup() {
pinMode(10, OUTPUT); // power up data logger
if (!SD.begin(10)) {
return;
}
pinMode(4,OUTPUT); // set LED pin to output
digitalWrite(4,LOW);
analogReference(DEFAULT); // will read analog inputs between 0 and 5 volts
myFile = SD.open("data.txt", FILE_WRITE); // specify name of text file on SD card
```

```
myFile.println("Start"); // prints initializer 'Start' at beginning of file
}
void loop() {
if (myFile) {
for (int j = 0; j < 50; j++) { // collect 50 measurements
delay(510000); // delay until next measurement
digitalWrite(4,HIGH);
delay(90000); // allow LED to warm up for 1.5 minutes
for (int x = 0; x < 10; x++) { // collect ten measurements
read1 = analogRead(0);
delay(100);
read2 = analogRead(0);
delay(100);
read3 = analogRead(0);
delay(100);
read4 = analogRead(0);
delay(100);
read5 = analogRead(0);
val = (read1+read2+read3+read4+read5)/5; // average 5 measurements taken 100ms apart
time_t t = now(); // next 28 lines write the date and time to the SD card
if(month(t) < 10) {
myFile.print(0);
}
myFile.print(month(t));
myFile.print("/");
if(day(t) < 10) {
myFile.print(0);
}
myFile.print(day(t));
myFile.print("/");
myFile.print(year(t));
myFile.print(" ");
if(hour(t) < 10) {
myFile.print(0);
}
myFile.print(hour(t));
myFile.print(":");
if(minute(t) < 10) {
myFile.print(0);
}
myFile.print(minute(t));
myFile.print(":");
if(second(t) < 10) {
myFile.print(0);
}
myFile.print(second(t));
myFile.print(" @ ");
myFile.println(val);
}
digitalWrite(4,LOW); // turn LED off
```

```
}
}
myFile.close(); // closes file
while(1) { // LED blinks when measurements are done
digitalWrite(4,HIGH);
delay(1000);
digitalWrite(4,LOW);
delay(2000);
}
}
```

© 2013 by the authors; licensee MDPI, Basel, Switzerland. This article is an open access article distributed under the terms and conditions of the Creative Commons Attribution license (http://creativecommons.org/licenses/by/3.0/).
